# Microbial infection risk predicts antimicrobial potential of avian symbionts

**DOI:** 10.3389/fmicb.2022.1010961

**Published:** 2022-11-21

**Authors:** Ester Martínez-Renau, Mónica Mazorra-Alonso, Cristina Ruiz-Castellano, Manuel Martín-Vivaldi, Antonio M. Martín-Platero, María Dolores Barón, Juan José Soler

**Affiliations:** ^1^Departamento de Ecología Funcional y Evolutiva, Estación Experimental de Zonas Áridas (CSIC), Almería, Spain; ^2^Departamento de Zoología, Universidad de Granada, Granada, Spain; ^3^Unidad Asociada (CSIC): Coevolución: Cucos, Hospedadores y Bacterias Simbiontes, Universidad de Granada, Granada, Spain; ^4^Departamento de Microbiología, Universidad de Granada, Granada, Spain

**Keywords:** antimicrobial activity, antibiotic-producing bacteria, birds, natural selection, symbiotic bacteria, uropygial gland, uropygial secretion

## Abstract

Symbiotic bacteria on animal hosts can prevent pathogenic bacterial infections by several mechanisms. Among them, symbiotic bacteria can indirectly enhance host’s immune responses or, directly, produce antimicrobial substances against pathogens. Due to differences in life-style, different host species are under different risks of microbial infections. Consequently, if symbiotic bacteria are somewhat selected by genetically determined host characteristics, we would expect the antimicrobial properties of bacterial symbionts to vary among host species and to be distributed according to risk of infection. Here we have tested this hypothesis by measuring the antimicrobial ability of the bacterial strains isolated from the uropygial-gland skin of 19 bird species differing in nesting habits, and, therefore, in risk of microbial infection. In accordance with our predictions, intensity and range of antimicrobial effects against the indicator strains assayed varied among bird species, with hole-and open-nesters showing the highest and the lowest values, respectively. Since it is broadly accepted that hole-nesters have higher risks of microbial infection than open nesters, our results suggest that the risk of infection is a strong driver of natural selection to enhance immunocompetence of animals through selecting for antibiotic-producing symbionts. Future research should focus on characterizing symbiotic bacterial communities and detecting coevolutionary processes with particular antibiotic-producing bacteria within-host species.

## Introduction

Current view of symbiotic microbiomes acknowledges the beneficial effect of microorganisms on their hosts, in opposition to the classical view of microorganisms as pathogens. Thus, symbiotic bacteria are essential for understanding the evolution and functioning of their animal hosts (McFall-Ngai, 2002; Archie and Tung, 2015; Sherwin et al., 2019). Some bacteria, for instance, allow animals to achieve better digestion and use of nutrients (Backhed et al., 2004), enhance the immune system (Umesaki et al., 1995), promote chemical communication (Ezenwa and Williams, 2014; Carthey et al., 2018; Maraci et al., 2018), or even trigger direct defenses against parasite and/or predator enemies (McFall-Ngai et al., 2013; Suzuki, 2017; Mazorra-Alonso et al., 2021). Indeed, animals using bacterial symbionts or their metabolites for self-protection against infections is widely spread within the animal kingdom (Currie et al., 1999; Brucker et al., 2008; Soler et al., 2008; Brownlie and Johnson, 2009; Soler et al., 2010). Birds, for instance, tend to use antimicrobial-producing bacteria growing in nest lining materials (i.e., feathers; Peralta-Sánchez et al., 2010, 2018; Ruiz-Castellano et al., 2016, 2019) or in their uropygial gland (Soler et al., 2008), to prevent pathogenic infection of embryos (Martín-Vivaldi et al., 2014), nestlings (Soler et al., 2017) or breeding adults (Ruiz-Rodríguez et al., 2009).

Birds use their uropygial gland secretion to preen their feathers, which also confers protection against pathogenic infections during reproduction (Soler et al., 2012). The secretion is mainly composed of monoester and diester waxes of aliphatic alcohols and fatty acids with antimicrobial activity (Jacob and Ziswiler, 1982). This could partially explain the detected negative associations between size of the uropygial gland of different bird species and their eggshell bacterial loads and hatching success (Soler et al., 2012). Interestingly, in recent years, evidence showing that the uropygial gland of birds hosts symbiotic bacteria is accumulating in the literature. Following the pioneering works in woodhoopoes (*Phoeniculus purpureus*) (Law-Brown and Meyers, 2003) and European hoopoes (*Upupa epops*) (Soler et al., 2008), symbiotic bacteria have been detected in the uropygial secretion of turkeys (*Meleagris gallopavo*) (Braun et al., 2016), great spotted woodpeckers (*Dendrocopos major*) (Braun et al., 2018), American barn owls (*Tyto furcata*) (Braun et al., 2018), Egyptian geese (*Alopochen aegyptiacus*) (Braun et al., 2018), dark-eyed juncos (*Junco hyemalis*) (Whittaker and Theis, 2016), great tits (*Parus major*) (Bodawatta et al., 2020) and house sparrows (*Passer domesticus*) (Videvall et al., 2021). Moreover, in hoopoes some of the bacteria isolated from the gland secretion produce bacteriocins active against a wide range of bacterial strains, including potential pathogens such as *Listeria monocytogenes*, *Staphylococcus aureus* and the feather degrading *Bacillus licheniformis* (Martín-Platero et al., 2006; Martín-Vivaldi et al., 2010; Ruiz-Rodríguez et al., 2013). Thus, it is possible that the previously described antimicrobial properties of the uropygial secretions of birds (Shawkey et al., 2003; Reneerkens et al., 2008) were, at least partially, mediated by their antibiotic-producing bacterial symbionts.

In birds, antibiotic-producing bacteria, have also been found in avian body feathers, the bill, brood patch and eggshells (Cook et al., 2005; Shawkey et al., 2005; Martín-Vivaldi et al., 2014; Peralta-Sánchez et al., 2014; Soler et al., 2014; Martínez-García et al., 2015; Soler et al., 2016; Javůrková et al., 2019). All these locations are spread with uropygial secretion during preening and, thus, it is possible that some of these antibiotic-producing bacteria come from those inhabiting the uropygial gland of birds (Martínez-García et al., 2015; Soler et al., 2016). Another non-exclusive possibility is that some of these bacteria do not come directly from the bacterial communities in the secretion, but rather the secretion acts as a nutritional substrate facilitating or driving the colonization and growth of antibiotic-producing bacteria on the avian integumentary structures. In other words, it is possible that, thanks to the uropygial secretion, birds cultivate antibiotic-producing bacteria not only in their gland, but also on their feathers, beak and skin. Those symbiotic bacteria would help animals to prevent infections and, thus, would complement or add to the defensive responses of the immune system (Soler et al., 2010). If this is the case, since antibiotic-producing bacteria development would depend on the bird’s physiology (e.g., by producing a large amount of uropygial secretion of special chemical characteristics), birds might be able to select the antimicrobial characteristics of their bacterial symbionts. In this scenario, as it occurs with the immune system, antimicrobial capacity of those bacteria should adjust to the expected level of risk of infection. Here, we explore some predictions to test this hypothesis in a comparative interspecific framework.

The general predictions of the hypothetical selection of antimicrobial capacities of bacterial symbionts according to risk of infection for hosts are: (i) antimicrobial capacity of isolated symbiotic bacteria should differ among species; and (ii) species-specific values of the antimicrobial potential should correlate with the strength of selection pressure, i.e., with the risk of infection experienced by each studied species. Life-style and life history strategies may condition risk of infection of animal populations. For instance, breeding in cavities entails high risks of ectoparasitism and pathogenic infections (Møller, 1997; Peralta-Sánchez et al., 2018). Secondary-cavity nesters are in some way obligated to re-use holes due to the scarcity of natural ones. This life-style facilitates parasite transmission and multiple infections by more virulent parasites (Wiebe et al., 2007; Møller et al., 2009), and, on the other hand, selects for more efficient immune systems (Møller and Erritzøe, 1996). Thus, if antimicrobial capacity of symbiotic bacteria is selected according to risk of infection of their hosts, we expect that those isolates from hole-nester species should be more efficient antagonizing the proliferation of other microorganims than isolates from non-hole nester species.

Immune responses also vary with age, either because of ageing of different components of the immune system (Cichon et al., 2003; Müller et al., 2013), or because juveniles have not yet fully developed (Palacios et al., 2009; Stambaugh et al., 2011). However, the effect of age depends on the immune parameters considered (Vermeulen et al., 2017). Nestlings typically show lower innate immune response (Ricklefs, 1992; Killpack and Karasov, 2012), but higher cell mediated immunity (Tella et al., 2002) than adults. As suggested above, birds could cultivate antibiotic-producing bacteria on their feathers, beak, and skin. The uropygial gland is not fully developed before fledging (Jacob and Ziswiler, 1982) and characteristics and quantity of secretion typically vary among nestlings of different ages and between nestlings and adults (Jacob and Ziswiler, 1982; Soler et al., 2022). Thus, due to age differences in immunity and in uropygial secretion properties, it can be predicted that the bacterial community associated with the uropygial secretion, and, thus, its antimicrobial capacity, varies with age too.

In this study, we characterized the antimicrobial capacity of bacterial isolates from the uropygial gland skin of nestlings and/or adults of 19 bird species against several referential bacterial strains. We then explore the intra-and interspecific variations in intensity and range of antimicrobial capacity, and tested the hypothesis that antimicrobial capabilities of microbial symbionts is adjusted to the risk of infections of their avian hosts with the expectations that they should vary according to species identity, age and nesting habits.

## Materials and methods

### Study area and species

Fieldwork was carried out during the breeding seasons of 2018 and 2019, in southern Spain; in the Hoya de Guadix (37°15′N; 3°01′W), a semiarid high-altitude plateau, and in the Charca de Suárez, a wetland near the coast in Motril (36° 43′ 18.707”N, 3° 32′ 30.836”W). Most nestlings and adults of hole-nester species were sampled during breeding in cork-made nest boxes (internal dimensions: 180 mm x 210 mm and 350 mm high, 240 mm from the bottom to the hole entrance) located in the Guadix study area, where we also sampled nestlings of open-nester species few days before leaving the nests. We used mist nets to capture adults or fledglings few days after abandoning the nest, mainly in the Charca de Suárez, but also in Guadix.

### Fieldwork

Since March 15th, we visited nest boxes every 7–8 days, and intensively looked for nests of non-hole nesters in the surroundings until egg detection, which allowed us to calculate the hatching date and, thus, planning the sampling date of adults and nestlings. Hole-nester adults were captured within the nest boxes during brooding, and nestlings were sampled during the last quarter of the nestling period. Briefly, we sampled the bacterial community of the uropygial gland skin by rubbing the surface area of the uropygial gland, including the opening, with a sterile cotton swab slightly wetted in sterile Phosphate Buffer Saline (PBS, 0.2 M). The surface of the sampled uropygial gland skin was estimated by multiplying the length and the width of the gland. The swab was kept in a sterile microfuge vial containing 1 ml of sterile PBS and stored at 4°C until further processing. We also measured tarsus length with a digital caliper (accuracy 0.01 mm), wing length with a ruler (accuracy 1 mm), body mass with a digital scale (accuracy 0.01 g), and gland dimensions (length, width and height) with a digital caliper (accuracy 0.01 mm) following Martín-Vivaldi et al. (2009). Finally, we ringed all individuals with numbered metal rings (Ministerio de Agricultura, Spain) to avoid resampling.

### Laboratory procedure

#### Isolation of bacteria from the uropygial gland skin

We processed the uropygial skin samples in the laboratory the same day of collection. After vortexing the microfuge tubes containing the swabs, we plated decimal dilutions up to 10^−4^ on Tryptic Soy Agar (TSA, Scharlau, Barcelona, Spain). Petri dishes were incubated aerobically for 24 h at 37°C. Bacterial counts were estimated by standardization of the number of colonies per cm^2^ of sampled uropygial gland skin.

#### Antimicrobial activity of colonies isolated from uropygial gland skin

We isolated five morphologically different colonies from each plate. To assure that a pure culture was achieved, each of these colonies were serially cultivated by the streak-plate method onto TSA plates for three times, incubating them for 24 h at 37°C. We then assayed their production of antimicrobial substances by the double-layer technique (Gratia and Fredericq, 1946) against 9 indicator bacterial strains. To this end, each isolate was replicated by spotting onto 9 TSA petri dishes (30 isolates per plate), and incubated for 24 h at 37°C before performing the antagonistic tests used to estimate antimicrobial capacity of sampled individuals. After producer bacteria were grown, plates were covered with 7 ml of soft agar (BHI added 0.8% agar, Scharlau Chemie S.A., Barcelona) previously heated until liquefied and tempered to 50°C. Once liquid, the soft agar was inoculated with 100 μl of an overnight culture of the indicator strain (see below) at 37°C. Finally, covered plates were incubated for 24 h at 37°C. The antimicrobial activity of each isolated colony was measured as the width of the inhibition halo around the spot of the colony, measured with a ruler to the nearest 0.5 mm. No control strains were used to standardize the halo width, then raw data was employed for statistical analyses [for more details see Ruiz-Rodríguez et al. (2012) and Ruiz-Castellano et al. (2019)].

The antimicrobial assays were performed against nine typified strains covering a wide range of bacterial taxa which include potential pathogenic bacteria for birds (Pinowski et al., 1994; Hubalek, 2004; Benskin et al., 2009). These strains come from the Spanish Type Culture Collection (CECT) and from our laboratory. We used *Bacillus licheniformis* D13, *Enterococcus faecalis* S47, *Escherichia coli* CECT774, *Listeria inocua* CECT340, *Micrococcus luteus* 241, *Mycobacterium* sp., *Pseudomonas putida, Salmonella choleraesuis* CECT443, and *Staphylococcus aureus* CECT240.

### Statistical analysis

We sampled 326 individuals from 19 species, including non-hole (N = 29 adults, 12 nests, 16 nestlings, Table 1) and hole nesters (N = 84 adults, 105 nests, 200 nestlings, Table 1). For eight of those species we collected information from both adults and nestlings close to fledging, for six species we only sampled nestlings, and for five species we only sampled adults. For each bacterial isolate, antimicrobial capacity was estimated as the average of the width of antagonistic halos (hereafter intensity of antimicrobial activity) when tested against each of the nine indicator bacteria. Moreover, we also estimated the diversity (Shannon index) of antimicrobial activity, which informs on the range of antimicrobial activity of each bacterial isolate. Then, for each individual, we averaged the antimicrobial activity and antimicrobial range values of the five isolates from its uropygial gland skin. Bacterial density of the uropygial gland skin, as well as intensity and range of the antimicrobial activity of bacteria isolated from individuals (i.e., nestlings) of the same nest, were consistent (R^2^ > 70%) and, then, we used nest mean values, which would appropriately account for the non-independence data of siblings. Bacterial density were log10 transformed to approach a normal distribution, while intensity and range of antimicrobial activity did not differ significantly from the Gaussian distribution.

**Table 1:** Sample sizes of adults, nests and nestlings sampled for each species of hole nester and non-hole nester species.

Species		Sample size		
		Nests	Nestlings	Adults
**Hole nesters**	Athene noctua	8	14	6
	Columba oenas	6	12	
	Coracias garrulus	8	15	3
	Corvus monedula	6	12	
	Otus scops	8	16	9
	Parus major	7	12	3
	Passer domesticus	8	16	11
	Petronia petronia			5
	Picus viridis	4	8	
	Pyrrhocorax pyrrhocorax	6	11	
	Sturnus unicolor	9	17	9
	Upupa epops	35	67	38
**Non-hole nesters**	Acrocephalus scripaceus			7
	Cettia cetti			4
	Chloris chloris			3
	Columba palumbus	3	5	
	Corvus corone	3	5	
	Muscicapa striata			4
	Serinus serinus	6	6	11

To explore the effects of age (nestling versus adult) and species identity on the intensity and the range of the antimicrobial activity of bacteria isolated from their uropygial gland (hereafter antimicrobial variables), and on bacterial density, we first used General Linear Models (GLMs). These models included age and species identity as fixed factors, while the interaction between these two factors was explored in separate models. Because antimicrobial variables consistently differed for adults and nestlings, (even after controlling the effect of species identity (see Results)), the effect of nesting habits and species identity (nested within nesting habits to control for the non-balance species-data) were explored in separate GLM models for adults and nestlings. This approach allows increasing the number of considered species from 8 species with information for adult and nestlings, to 14 and 13 species, respectively.

Furthermore, we also explored the effects of nesting behavior and species identity on the antimicrobial profile of bacteria isolated from the uropygial gland skin of birds. To do this, we first calculated average individual/nest values of intensity of antimicrobial activity against each of the indicator bacterium used, and later estimated distance matrices among sampled individuals/nests based on the Bray–Curtis similarity measure. Then, we explored the effects of fixed factors (age, nesting habits and species identity) on the distance matrix by means of PERMANOVAs as implemented in Primer7 v.7.0.17 (PRIMER-e). Similarly to the approach described for GLMs, we first checked the effect of age on the antimicrobial profile of the eight species with information for both nestlings and adults. Since we found a strong effect of age even after controlling for the effect of species identity (see Results), subsequent models directed to explore the effects of nesting habits were computed independently for adults and nestlings. These PERMANOVAs included nest type and species identity nested within nest type as independent fixed factors. Principal Coordinates Analyses (PCoA) were used to visualize the relative position of species centroids (± 90% CI of ellipses) in the multidimensional space.

Finally, as nesting habits have a strong phylogenetic component, we tried to control the analyses for phylogeny by means of Bayesian phylogenetic mixed models (MCMCglmm). First, we downloaded 100 trees for our set of species from http://birdtree.org/ [source of trees was Ericson all species; (Jetz et al., 2012)] and estimated the predicted effects for each of the trees using the MCMCglmm package (Hadfield, 2010) in R (R-Core-Team, 2020) environment that also included the packages “ape” (Paradis et al., 2004), “MASS” (Paradis et al., 2004) and “mvtnorm” (Venables and Ripley, 2002). To run the model we used the uninformative prior [list(G = list(G1 = list(V = 1,nu = 0.002)),R = list(V = 1,nu = 0.002)], and adjusted the number of iterations, to 100,000, the burn-in period to 10,000 and the thinning interval to 10. That model was run for each of the 100 trees and calculated average values and the minimum and maximum values of lower and upper 95% credibility intervals of estimates. We also used Geweke’s convergence diagnostic for Markov chains (Geweke, 1992), which is based on a standard *z*-score of means of the first (10%) and the last part (50%) of a Markov chain. These z-scores never exceeded the critical value of 1.96. The random effect of phylogeny is reported as heritability (h2) (Hadfield, 2010), which is a measure of phylogenetic signal analogous to Pagel’s lambda that ranges from zero (non-phylogenetic signal) to one (high phylogenetic signal).

## Results

### Age effects

**Figure 1:**
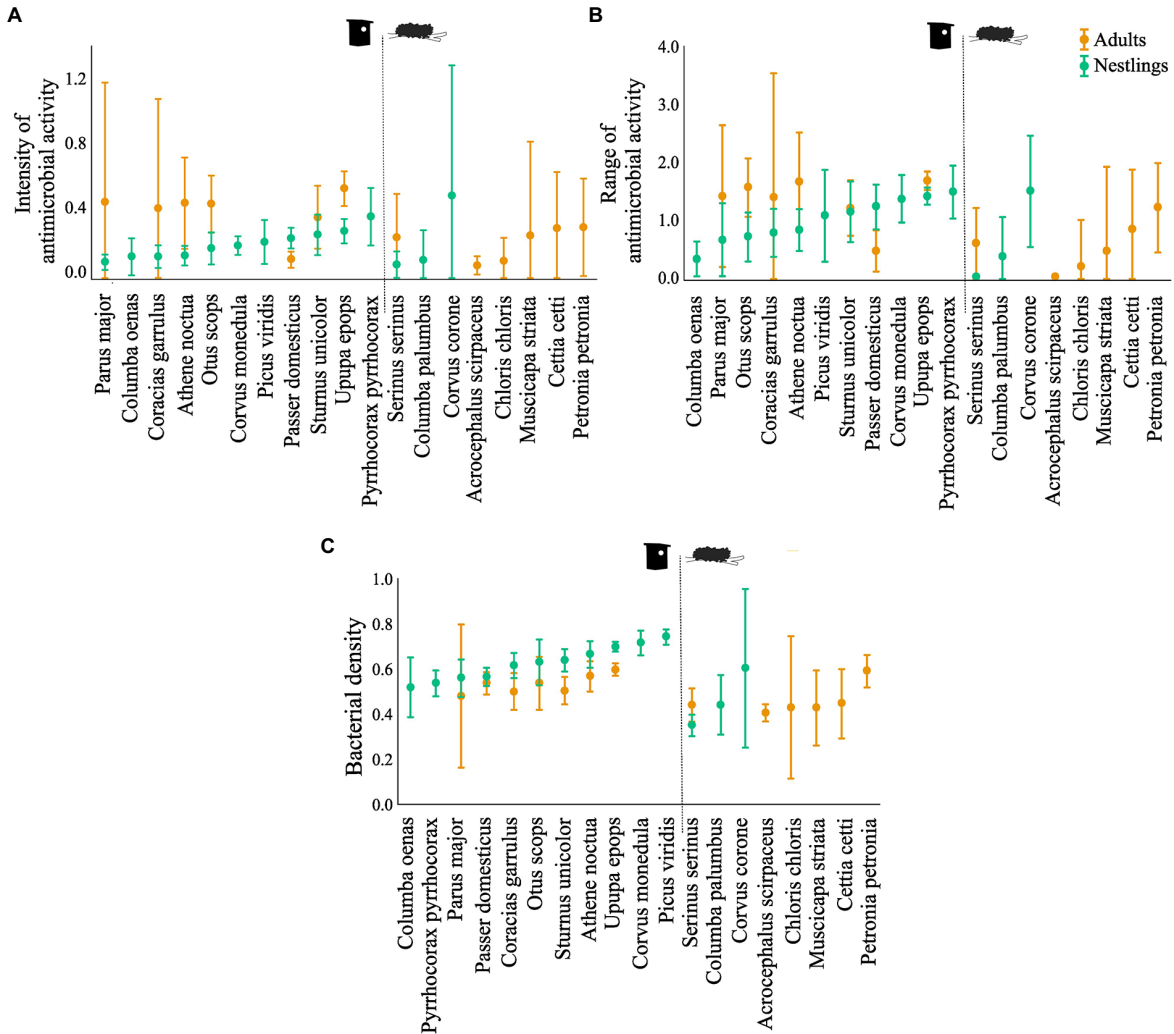
Antimicrobial capacity: **(A)** antimicrobial activity (average values of the width of antagonistic halos (mm) when tested against each of the nine indicator bacteria) and **(B)** antimicrobial range (Shannon index of the antimicrobial activity), of the bacterial communities associated to the uropygial gland skin of different bird species. **(C)** Bacterial density on the gland surface of the same species. Results for adults and nestlings are shown in different colours. Values are means ±95% CI. CI are not symmetric because negative values are not possible.

Bacterial isolates from the uropygial gland skin of adults demonstrated higher intensity and range of antimicrobial activity than those from nestlings, although the effect depended on species identity (Table 2; Figure 1). Moreover, density of bacteria on the uropygial gland skin was higher in nestlings than in adults, although it again depended on species identity (Table 2; Figure 1).

**Table 2:** Results from General Linear Models (GLM) and PERMANOVAs with antimicrobial activity (average values of the width of antagonistic halos when tested against each of the nine indicator bacteria), antimicrobial range (Shannon index of the antimicrobial activity), bacterial density and the antimicrobial profile as dependent variables.

	General Linear Models (GLM)									PERMANOVA		
	Antimicrobial **activity**			Antimicrobial **range**			**Bacterial density**			Antimicrobial **profile**		
	*F*	*df*	*P*	*F*	*df*	*p*	*F*	*df*	*p*	Pseudo-F	*df*	*p*
Species	4.14	8,173	**<0.001**	9.17	8,173	**<0.001**	6.89	8,164	**<0.001**	3.7	8,165	**<0.001**
Age	35.19	1,173	**<0.001**	12.87	1,173	**<0.001**	52.97	8,164	**<0.001**	9.56	1,165	**<0.001**
Species*Age	1.86	8,165	0.07	3.7	8,165	**<0.001**	2.89	8,156	**0.005**	1.61	8,165	**0.003**

Species identity and age (nestlings vs. adults) were used as the independent factors. The interaction between species identity and age was explored in separate models that also included main effects. Values in bold are statistically significant.

### Nesting habits effects on adults and nestlings

**Figure 2:**
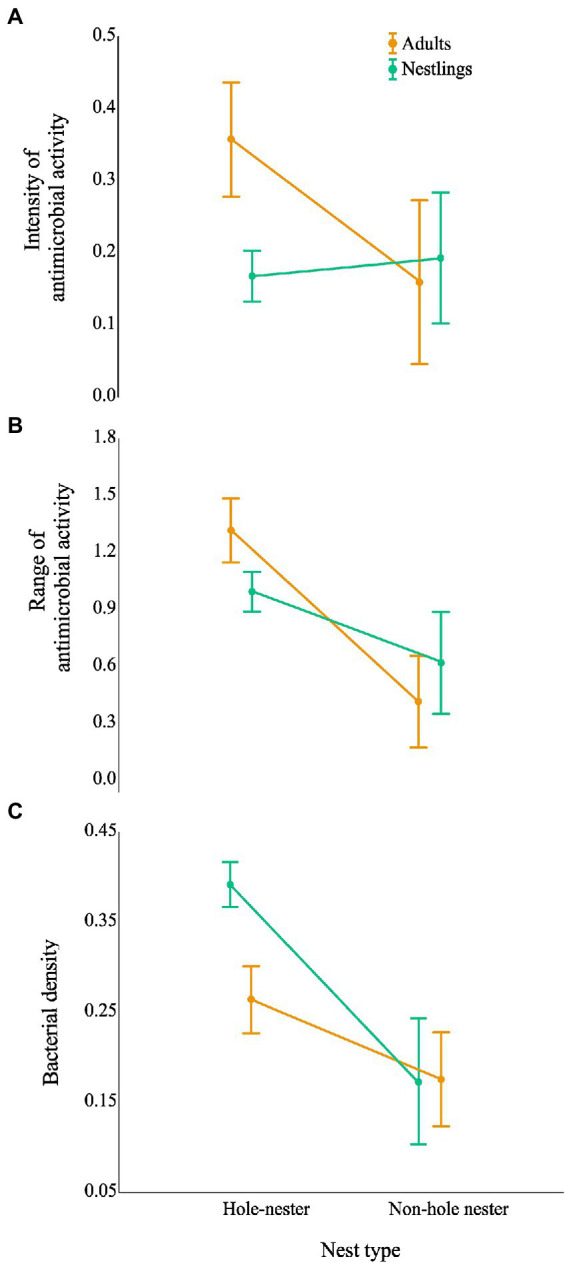
Effect of nesting habits of birds (hole *vs* non-hole) on the antimicrobial capacity and bacterial density of the community living on their uropygial gland skin. Graphs show the estimated antimicrobial capacity: **(A)** intensity of antimicrobial activity (average values of the width of antagonistic halos (mm) when tested against each of the nine indicator bacteria) and **(B)** antimicrobial range (Shannon index of the antimicrobial activity), as well as **(C)** density of bacteria isolated from the birds’ gland skin after controlling for species identity nested within nest type. Results for adults and nestlings are shown in different colours. Values are least square means ±95% CI.

**Figure 3:**
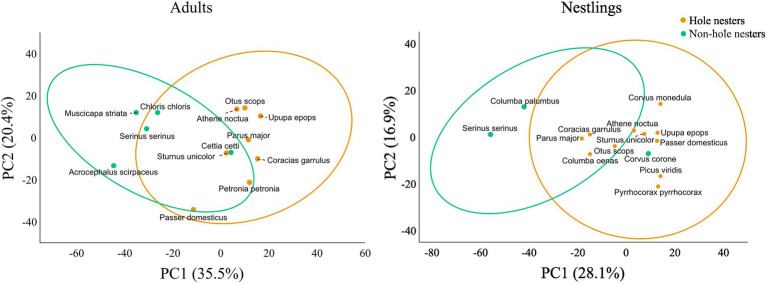
Principal Coordinates Analisis (PCoA) ordination plot using bray-curtis distances of the antimicrobial profile of bacterial communities isolated from the uropygial gland skin. Points are centroids per species and ellipses indicate 90% CI.

Although total bacterial density of the uropygial gland skin of adults did not differ significantly among species, intensity and range of antimicrobial activity of bacterial isolates did (Table 3). As expected, adults of species with bacterial symbionts of higher intensity and range of antimicrobial activity [the hoopoe (*Upupa epops*), the great tit (*Parus major*), the little owl and the Eurasian scops owl (*Otus scops*)] were hole-nesters, while those with bacterial symbionts with lower values [reed warblers (*Achrocephalus scirpaceus*) and green finches (*Chloris chloris*)] were non-hole nesters (Figure 1). Thus, on average, antimicrobial capacity of symbiotic bacteria isolated from adults of hole-nester species were more intense and diverse than those isolated from non-hole nesters (Figure 2; Table 3). Similarly, when considering compositional variance of the antimicrobial profile, they significantly differed among species, and between hole-nester and non-hole-nester (Table 3; Figure 3). Interestingly, bacterial density on the skin of adults depended on nesting habits, but not on species identity within hole and non-hole nester groups (Table 3). The bacterial density on uropygial gland skin of hole-nester species was higher than that on non-hole nesters (Figure 2), suggesting that hole-nester species experience higher risk of bacterial infection. Phylogenetic corrected analyses confirmed all those results, but differences in intensity of antimicrobial activity of bacteria isolated from hole- and non-hole-nester species was only close to statistical significance (Table 4). Interestingly, the three models showed significant phylogenetic components suggesting that antimicrobial capacity of bacteria from close-related species is similar to each other.

**Table 3:** Results of nested General Linear Models (GLM) and PERMANOVAs exploring the effects of nest type (hole vs. non-hole) and species identity (nested withing nest type) on the antimicrobial activity (average values of the width of antagonistic halos when tested against each of the nine indicator bacteria), antimicrobial range (Shannon index of the antimicrobial activity), bacterial density and the antimicrobial profile in separated models for each of the dependent variable, and for adults and nestlings.

	**Adults**			**Nestlings**		
	F*	*df*	*p*	F*	*df*	*p*
*Antimicrobial activity*						
Nest type	8.02	1,97	**0.006**	0.26	1,104	0.609
Species (Nest type)	2.45	11,97	**0.010**	4.34	12,104	**<0.001**
*Antimicrobial range*						
Nest type	36.74	1,97	**<0.001**	6.69	1,104	**0.011**
Species (Nest type)	4.32	11,97	**<0.001**	6.47	12,104	**<0.001**
*Total bacterial density*						
Nest type	23.14	1,93	**<0.001**	33.98	1,104	**<0.001**
Species (Nest type)	1.51	11,93	0.142	5.21	12,104	**<0.001**
*Antimicrobial profile*						
Nest type	8.54	1,97	**<0.001**	5.74	1,104	**<0.001**
Species (Nest type)	2.18	11,97	**<0.001**	1.7	12,104	**<0.001**

*F statistic is Pseudo-F in PERMANOVA analysis for antimicrobial profile. Values in bold are statistically significant.

**Table 4:** Results of MCMCglmm models with nest type as a fixed factor, the bird phylogeny as a random factor and the antimicrobial activity (average values of the width of antagonistic halos when tested against each of the nine indicator bacteria), antimicrobial range (Shannon index of the antimicrobial activity), and bacterial density as dependent variables.

		Lower 95% CI	Upper 95% CI	ESS Lower 95% CI	ESS Upper 95% CI	Autocorr Lower 95% CI	Autocorr Upper 95% CI	(z-score) Lower 95% CI	(z-score) Upper 95% CI	pMCMC (−95%CI)	pMCMC (+95%CI)
Adults	*Antimicrobial activity*										
	Nest type	−0.374	0.018	8960.682	9120.342	−0.002	0.002	−0.337	0.049	0.072	0.074
	heritability	0.012	0.354								
	*Antimicrobial range*										
	Nest type	−1.358	−0.288	9012.492	9124.574	−0.004	0	−0.308	0.054	**0.006**	**0.006**
	heritability	0.07	0.53								
	*Bacterial density*										
	Nest type	−0.164	−0.002	8966.204	9061.441	−0.001	0.003	−0.254	0.2	**0.045**	**0.047**
	heritability	0.012	0.28								
Nestlings	*Antimicrobial activity*										
	Nest type	−0.128	0.155	8839.062	9037.088	0	0.005	−0.109	0.25	0.876	0.886
	heritability	0.034	0.514								
	*Antimicrobial range*										
	Nest type	−0.805	0.205	8948.682	9050.966	−0.002	0.002	−0.119	0.275	0.222	0.227
	heritability	0.178	0.68								
	*Bacterial density*										
	Nest type	−0.314	−0.065	8932.945	9032.842	−0.001	0.003	−0.319	0.097	**0.006**	**0.006**
	heritability	0.17	0.705								

The average of the 100 trees was calculated for each factor, as well as the 95% CI calculated as the lower and upper values for 95% Credibility Intervals of each estimate. Values in bold are statistically significant.

Similar to the results for adults, we found significant interspecific differences in bacterial density, and in the intensity, range and profile of antimicrobial capacity of bacteria isolated from the skin of the uropygial gland of nestlings (Table 3; Figures 2, 3). In this case, the species with bacterial symbionts of higher intensity and range of antimicrobial activity [the carrion crow (*Corvus corone*)] was a non-hole nester, although it was followed by two hole-nester species; the red-billed chough (*Pyrrhocorax pyrrhocorax*) and the hoopoe. Moreover, two out of three species with bacterial symbionts with lower values of intensity of antimicrobial activity and of range of antimicrobial capacity were non-hole nesters (Figure 1). Thus, although differences among hole and non-hole nesting species followed similar pattern in nestlings and adults, the intensity of antimicrobial activity of bacterial symbionts isolated from the gland skin of nestlings of hole- and non-hole-nester species did not differ significantly (Table 3; Figure 2). In any case, similarly to what we found in adults, estimated antimicrobial range (Figure 2) and antimicrobial profiles (Figure 3) of nestlings of hole- and non-hole nester species differed significantly (Table 3). Moreover, antimicrobial range, and bacterial density, were of higher values in hole-nester species (Table 3; Figure 2). Phylogenetic corrected analyses only confirmed detected differences in bacterial density, while all three models showed significant phylogenetic components (Table 4).

## Discussion

In the present study we examined the antimicrobial capacities of the bacterial community harbored on the skin of the uropygial gland of nestlings and adults of hole- and non-hole-nester bird species. Our results reveal that, although the effects of age and nesting habits depended on the species identity, antimicrobial capacity of bacteria isolated from adults were more intense and diverse than those of bacteria isolated from nestlings. The uropygial skin of nestlings, however, carried bacteria at a higher density than that of adults. Moreover, intensity and range of antimicrobial capacities of bacterial isolates were higher in hole-nester species, which also harbored bacteria at a higher density. These effects of nesting habits were mainly detected for adult birds. Finally, variables describing either antimicrobial capacities or bacterial density of different bird species have a moderate but significant phylogenetic component. Because adult birds might have a more stable bacterial community than nestlings, and hole-nesters are under higher risk of bacterial infection than non-hole nesters are, those results might suggest that natural selection favors the establishment and growth of competent antibiotic-producing bacteria on the skin of birds. Below, we discuss that possibility and the potential role of uropygial secretion selecting antibiotic-producing bacteria that would contribute to the bird’s antimicrobial defenses.

We detected consistent among-species differences in characteristics of the bacterial communities, which occurred even after controlling for the effect of age or nesting habits. These differences could be explained by different species exploiting different resources or habitats where the pool of bacteria able to colonize the skin of birds might also differ (Thompson et al., 2017). Bacterial communities of holes and open habitats that birds use for breeding differ (Godard et al., 2007; Peralta-Sánchez et al., 2012) and, thus, skin bacterial communities of hole and non-hole nesters should accordingly differ. Although our results fit that prediction in terms of bacterial density, it is important to highlight that bacteria from hole-nester species consistently demonstrated higher intensity and range of antimicrobial activity, which could also be explained because of a higher competition among bacterial strains in environments with higher bacterial densities, such as the gland skin of hole-nester species, thus favoring the establishment of antibiotic-producing bacteria.

Another non-exclusive explanation is that different species might differ in mechanisms preventing bacterial colonization of skin and some other integuments of birds (Soler et al., 2011; Peralta-Sánchez et al., 2018; Javůrková et al., 2019; Azcárate-García et al., 2020) that would result in species-specific skin bacterial communities with particular antimicrobial properties. Appropriate holes for bird breeding are scarce in nature (Newton, 1994) and, thus, they are likely reused from season to season (Aitken et al., 2002; Wiebe et al., 2007). Interestingly, nest reuse increases the risk of horizontal transmission of ectoparasites and pathogens (Møller and Erritzøe, 1996; Tomás et al., 2007) and density of bacteria in nestlings’ skin (González-Braojos et al., 2012). In accordance, we found higher bacterial density in the uropygial gland skin of hole-nester than in that of non-hole-nester species. Importantly, bacterial density of avian nests predicts the probability of hatching failure (Peralta-Sánchez et al., 2018), and, thus, selection pressures favoring the evolution of antibacterial defenses [i.e., innate humoral immunity (Soler et al., 2011)] should be stronger for hole-nester species. Accordingly, the size of the bursa of Fabricius and the spleen of these species is larger than that of non-hole-nester species (Møller and Erritzøe, 1996). Here, fitting with this pattern, we found that the antimicrobial capacity of bacteria isolated from hole-nester species were higher than that of non-hole nesters isolates. However, to conclude in favor of the hypothesis that our findings are the consequence of natural selection, a mechanism resulting from bird phenotypes must first be demonstrated as the cause of the detected differences in antimicrobial properties of bacteria isolated from hole-nester and non-hole-nester species.

A third non-exclusive possibility explaining interspecific differences in antimicrobial properties of bacteria isolated from the skin of the uropygial gland of birds is that characteristics of the uropygial secretion, which differ among species (Jacob and Ziswiler, 1982), are associated with interspecific variation of bacterial communities (Soler et al., 2012). Uropygial secretion might thus be the avian trait where natural selection works favoring antimicrobial potential of the microbial symbionts. Birds preen their feathers, bills and skin with uropygial secretion and, thus, the detected interspecific effects might be due to interspecific differences in antimicrobial properties of secretion (Moreno-Rueda, 2017). Moreover, the preen secretion of different species also varies in chemical composition (Jacob and Ziswiler, 1982) that might differentially enhance the growth of particular bacteria explaining detected interspecific variation in bacterial communities. Interestingly, chemical composition of the uropygial secretion of birds typically changes with phenology. The uropygial secretion of hoopoe females, for instance, changes during reproduction as does its antimicrobial capabilities (Soler et al., 2008), which is mediated by the microbial symbionts (Ruiz-Rodríguez et al., 2009). Thus, although we know that microbial symbionts affect the chemical composition of secretion (Martín-Vivaldi et al., 2010), it is plausible that the remarkable changes in chemical composition of uropygial secretions command colonization and growth of their antibiotic-producing bacteria. Similarly, it is also possible that, because birds use the uropygial secretion for preening, interspecific variation in chemical composition of the secretion was the cause of the detected interspecific variation in antimicrobial capability of microbial symbionts. It has also been suggested that uropygial secretion modulated the microbiota of body feathers (Jacob et al., 2018), which might suggest that the uropygial secretion is also responsible for the detected antimicrobial characteristics of the bacteria that we isolated from the skin of the uropygial gland of different species.

Apart from its antimicrobial properties, the uropygial secretion of birds might function in scenarios of chemical communication including that related to inadvertent social information (Danchin et al., 2004; Caro et al., 2015; Mazorra-Alonso et al., 2021). Accordingly, variations in chemical composition have been described in association with individual characteristics including age, sex, and phenology (Reneerkens et al., 2002; Leclaire et al., 2011a,b; Amo et al., 2012a; Díez-Fernández et al., 2021). Here, we have detected that the antimicrobial capacity of bacterial isolates differed depending on age, which might also be interpreted as a consequence of the age related variation of the chemical composition of the uropygial secretion between adults and nestlings. Age differences, however, would be hardly explained as a consequence of selection pressures due to risk of infections, mainly because bacteria grew at a larger density in nestlings than in adults, but also because antimicrobial activity of bacteria isolated from nestlings skin of hole-nester species did not differ from that of non-holer species. However, in accordance with the hypothetical role of natural selection driving antimicrobial capacity of nestling symbionts, the antimicrobial profile of nestlings of holer- and of non-holer species differed significantly, with antimicrobial range of nestlings of the former species being larger than that of the latter group of species. Similarly to the age effects detected in antimicrobial capacity of bacterial symbionts, the immune responses of nestling birds are also typically weaker than that of adults (see Introduction), which is mainly explained as an ontogenetic effect of developing immune system (Apanius, 1998). Thus, it is possible that the detected age effect was also due to the ontogenetic effects of developing uropygial secretion responsible of known differences in characteristics of the uropygial secretion of birds (Amo et al., 2012a,b; Soler et al., 2022); a possibility worth exploring in the future.

Summarizing, we detected parallelism in the antimicrobial capacities of microbial symbionts and the strength of selection due to parasitic infections associated with nesting habits. Because detected differences could be hardly explained by random effects, we suggest that natural selection should favor mechanisms (i.e., characteristics of uropygial secretion) allowing cultivation of antibiotic-producing bacteria on the uropygial glands skin, feathers and other birds’ teguments. These results therefore suggest a new line of animal immunity mediated by natural selection acting on traits determining antimicrobial capacity of their bacterial symbionts. Future research should focus on characterizing symbiotic bacterial communities and detecting coevolutionary processes with particular antibiotic-producing bacteria within-host species.

## Data availability statement

Data used in this paper can be found in CSIC Institutional Repository: https://doi.org/10.20350/digitalCSIC/14748.

## Ethics statement

The animal study was reviewed and approved by the Environmental Department of the Regional Government of Andalucía, Spain (reference SGYB/FOA/AFR).

## Author contributions

JJS and MM-V: conceived and designed the experiments. EM-R, MM-A, CR-C, MB, JJS, and MM-V: fieldwork. AMM-P, CR-C, EM-R and MB: lab work. JJS and EM-R: analyzed the data. EM-R wrote the first version with the supervision of JJS. All the authors substantially contributed reagents, materials, analysis, tools, and contributed to the final version of the manuscript.

## Funding

EM-R was financed by a predoctoral contract (PRE2018-085378) while the whole research group received funds from the projects CGL2017-83103-P, PID2020-117429GB-C21, and PID2020-117429GB-C22, funded by the Ministerio de Ciencia e Innovación/Agencia Estatal de Investigación/10.13039/501100011033 and by “Fondo Europeo de Desarrollo Regional, a way of making Europe.” The research group also benefits from facilities, including accommodation, provided by the City Hall of Guadix, where a small lab to quickly process the samples was installed.

## Conflict of interest

The authors declare that the research was conducted in the absence of any commercial or financial relationships that could be construed as a potential conflict of interest.

## Publisher’s note

All claims expressed in this article are solely those of the authors and do not necessarily represent those of their affiliated organizations, or those of the publisher, the editors and the reviewers. Any product that may be evaluated in this article, or claim that may be made by its manufacturer, is not guaranteed or endorsed by the publisher.
